# Variations in Mre11/Rad50/Nbs1 status and DNA damage-induced S-phase arrest in the cell lines of the NCI60 panel

**DOI:** 10.1186/1471-2407-11-206

**Published:** 2011-05-27

**Authors:** Kristen M Garner, Alan Eastman

**Affiliations:** 1 Department of Pharmacology and Toxicology, and Norris Cotton Cancer Center, Dartmouth Medical School, Lebanon, NH 03756, USA

## Abstract

**Background:**

The Mre11/Rad50/Nbs1 (MRN) complex is a regulator of cell cycle checkpoints and DNA repair. Defects in MRN can lead to defective S-phase arrest when cells are damaged. Such defects may elicit sensitivity to selected drugs providing a chemical synthetic lethal interaction that could be used to target therapy to tumors with these defects. The goal of this study was to identify these defects in the NCI60 panel of cell lines and identify compounds that might elicit selective cytotoxicity.

**Methods:**

We screened the NCI60 panel in search of cell lines that express low levels of MRN proteins, or that fail to arrest in S-phase in response to the topisomerase I inhibitor SN38. The NCI COMPARE program was used to discover compounds that preferentially target cells with these phenotypes.

**Results:**

HCT116 cells were initially identified as defective in MRN and S phase arrest. Transfection with Mre11 also elevated Rad50 and Nbs1, and rescued the defective S-phase arrest. Cells of the NCI60 panel exhibited a large range of protein expression but a strong correlation existed between Mre11, Rad50 and Nbs1 consistent with complex formation determining protein stability. Mre11 mRNA correlated best with protein level suggesting it was the primary determinant of the overall level of the complex. Three other cell lines failed to arrest in response to SN38, two of which also had low MRN. However, other cell lines with low MRN still arrested suggesting low MRN does not predict an inability to arrest. Many compounds, including a family of benzothiazoles, correlated with the failure to arrest in S phase. The activity of benzothiazoles has been attributed to metabolic activation and DNA alkylation, but we note several cell lines in which sensitivity does not correlate with metabolism. We propose that the checkpoint defect imposes an additional mechanism of sensitivity on cells.

**Conclusions:**

We have identified cells with possible defects in the MRN complex and S phase arrest, and a series of compounds that may preferentially target S phase-defective cells. We discuss limitations of the COMPARE program when attempting to identify compounds that selectively inhibit only a few cell lines.

## Background

Many anticancer agents kill cells as a consequence of damaging their DNA. While this approach targets rapidly proliferating cells, it is only somewhat selective for tumors and can lead to undesirable toxicity to the patient. Differences exist between normal and tumor cells with respect to the cellular response to DNA damage and it may be possible to exploit these differences to selectively target tumor cells. One such difference is in cell cycle checkpoint regulation. In response to DNA damage, cell cycle checkpoints are activated causing the arrest of cell cycle progression [1]. This allows the cell time to repair the damage, or if too much damage has occurred, undergo cell death. Defects in checkpoints lead to mutations and cancer, and this genomic instability is now considered a characteristic of tumors [2]. As the checkpoints function to protect the cells from damage, it is hoped that their selective defect in tumor cells may result in sensitivity to novel therapeutic strategies.

The S-phase checkpoint is activated in response to DNA damage that occurs during replication. For example, topoisomerase I inhibitors such as SN38 produce a single-strand break in DNA which is converted to a double-strand break upon collision with the replication fork [3]. The MRN complex, consisting of Mre11, Rad50 and Nbs1, is recruited to the double-strand break and initiates the S-phase checkpoint by recruiting ataxia-telangiectasia-mutated (ATM) to the damaged site [4]. Processing at the break by the Mre11 nuclease generates regions of single-stranded DNA that recruit the ATM- and Rad3-related kinase (ATR) [5]. Activated ATR and ATM phosphorylate the checkpoint kinases Chk1 and Chk2, respectively, which in turn phosphorylate CDC25A targeting it for degradation and effectively arresting DNA synthesis through inactivation of the Cdk2/Cyclin A complex [6,7].

Mutations in the MRN complex have been identified in lymphoid, breast and colorectal tumors [8,9]. A defect in the MRN complex has also been identified in the colorectal tumor cell line, HCT116 [9]. Such mutations result in radioresistant DNA synthesis (RDS) and hypersensitivity to ionizing radiation [10-12]. In addition, MRN mutations have been reported to increase sensitivity towards anticancer drugs, such as topoisomerase I inhibitors and cisplatin [13,14].

Studies in our laboratory have confirmed that HCT116 cells have very low levels of Mre11 and also that they arrest poorly in S phase in response to SN38. By transfecting HCT116 cells with a vector expressing Mre11, we show here that we can reconstitute the MRN complex. Our data indicate that Mre11 is necessary for the stability of Rad50 and Nbs1 and rescues the S-phase arrest in HCT116.

MRN defects if present in some tumors could render these cells sensitive to DNA-damaging agents and thereby provide a tumor selective therapy. The primary goal of these studies was to find compounds that preferentially target cells with defects in the proteins of the MRN complex. We have examined the frequency of MRN defects across the NCI60 cell line panel which is derived from many different cancer types (albeit only 59 are presently available) [15]. These cell lines have been characterized pharmacologically by exposure to more than 100,000 compounds (http://dtp.nci.nih.gov). We then correlated the MRN protein levels to sensitivity to the compounds in the NCI database using the COMPARE program [16,17]. Since there are many other proteins involved in the S-phase checkpoint we also assessed the cell lines for their ability to arrest in S phase when incubated with SN38, and then searched the database for additional correlations.

## Methods

### Cell culture

NCI60 cancer cell lines were obtained from the Developmental Therapeutics Program at the National Cancer Institute and were cultured in RPMI 1640 media (Hyclone) supplemented with 10% heat-inactivated fetal bovine serum (Hyclone) and Antibiotic-antimycotic (Gibco). Cells were cultured at 37°C with 5% CO_2_.

Elevated expression of Mre11 was achieved by cloning Mre11 from pDB20 (obtained from John Petrini, Memorial Sloan-Kettering Cancer Center) into pCR3.1 (Invitrogen) [18]. This plasmid was transfected into HCT116 cells using lipofectamine (Invitrogen), according to the manufacturer's protocol. Cells were selected in G418 (500 μg/ml) and multiple colonies were screened for maximum expression.

### Drugs and chemicals

SN38, the active metabolite of irinotecan, was obtained from Pfizer, and 2-(4-amino-3-methylphenyl)-5-fluorobenzothiazole (5F 203, NSC 703786) was obtained from the Developmental Therapeutics Program at the National Cancer Institute (Bethesda, MD). Both drugs were dissolved in DMSO and stored at -20°C.

### Flow cytometry

For cell cycle analysis, cells were rinsed with phosphate-buffered saline (PBS), harvested with 0.05% trypsin, and fixed in 70% ethanol overnight. Cells were stained with 100 μg/ml propidium iodide in 1 mg/ml ribonuclease A and analyzed on a FACScan flow cytometer (Becton Dickinson).

### Immunoblotting

Following treatment, cells were rinsed with phosphate buffered saline, lysed by direct addition of Laemmli buffer to the wells, boiled for 5 min, and stored at -80°C. Protein levels were quantified using EZQ Protein Quantitation Kit (Molecular Probes). 15 μg of each lysate was separated by SDS-PAGE, and transferred to polyvinylidene difluoride membranes. Membranes were blocked and incubated overnight at 4°C with primary antibodies specific for Nbs1 (Cell Signaling), Rad50 (Novus Biologicals), Mre11 or actin (Calbiochem). Membranes were washed and incubated for 30 min at room temperature with anti-rabbit or anti-mouse secondary antibody conjugated to horseradish peroxidase. Proteins were visualized with enhanced chemiluminescence. Densitometry scans were quantified using Scion Image software (Scion Corporation) and normalized to a serial dilution of the same protein from MDA-MB-231 present on each blot. Several exposures of each western blot were scanned to avoid over-exposure and to ensure that the samples were within the range of the standards.

### Cell growth assays

Cells were seeded at low density (500-1000 cells) in 96-well plates and then incubated with drug for 24 h. Following treatments, cells were washed and grown in fresh media for 5-7 days at 37°C. Prior to attaining confluence, cells were washed, lysed, and stained with Hoechst 33258, as previously described [19]. Fluorescence was read on a microplate spectrofluorometer (Spectramax M2). Within each experiment, each treatment was repeated in at least eight wells. Sensitivity to the "NSC" compounds was determined by the Developmental Therapeutics program at NCI and the data were obtained from their web site (http://dtp.nci.nih.gov). In their screen, all cell lines were incubated with a range of drug concentrations, usually for 48 h, and then fixed and stained with sulforhodamine B.

## Results

### Mre11 rescues S-phase arrest in HCT116 cells

**Figure 1:**
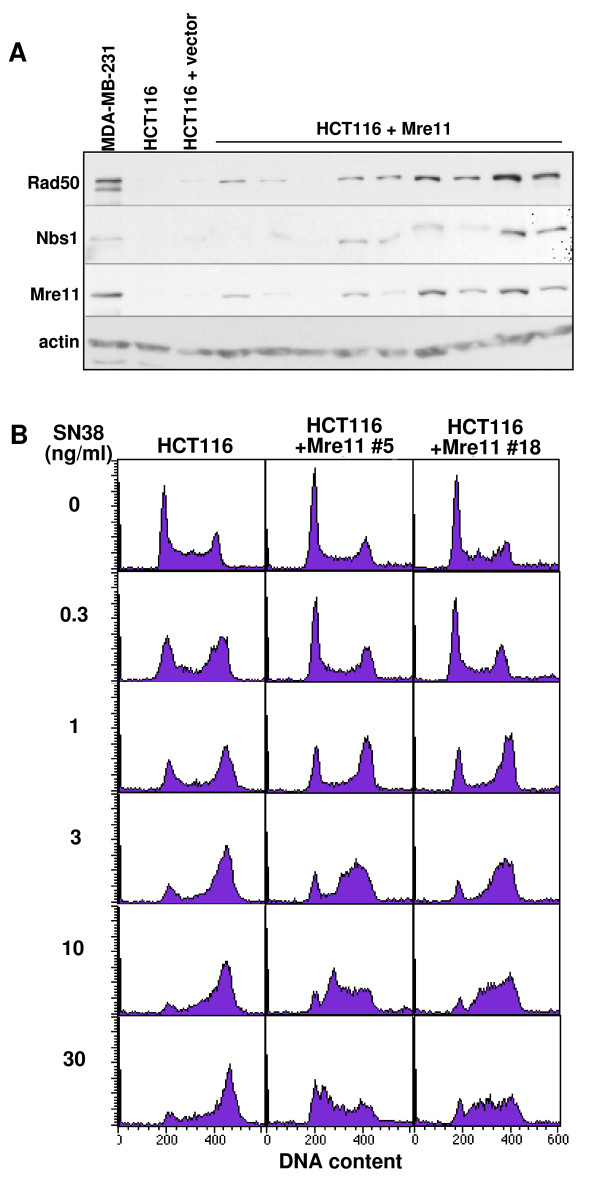
**Expression of Mre11 rescues S-phase arrest in HCT116**. HCT116 cells were transfected with a vector expressing Mre11 or an empty control vector and stable clones were selected. (A) Cells were lysed and Mre11, Rad50, Nbs1, and actin proteins were analyzed by western blot. The nine lanes on the right are derived from 9 independently-transfected clones. (B) HCT116 cells and two HCT116+Mre11 clones were incubated with 0-30 ng/ml SN38 for 24 h. Cells were fixed, stained with propidium iodide and analyzed for cell cycle distribution by flow cytometry.

DNA damage, such as that caused by the topoisomerase I inhibitor SN38, triggers checkpoints that arrest cell cycle progression to allow for DNA repair. We have previously compared seven cell lines for their ability to arrest in S phase [20]. In general, most cell lines arrest in late, middle or early S phase as the SN38 concentration increases. However, we found that HCT116 cells were notably different in that they showed primarily G2 arrest at high concentrations of SN38. Other studies have demonstrated that HCT116 cells have low levels of the MRN complex [9]. We confirmed that these cells have very low expression of Mre11 protein compared to MDA-MB-231 cells, as measured by western blotting (Figure 1). Rad50 and Nbs1 levels were also low in HCT116. Since Mre11 has been shown to be involved in the S-phase checkpoint, we hypothesized that the Mre11 defect was responsible for the lack of S-phase arrest in HCT116 cells. To test this hypothesis, we transfected HCT116 cells with a vector expressing Mre11. We selected individual colonies that expressed higher levels of Mre11 protein over control or vector-transfected cells (Figure 1). HCT116+Mre11 cells not only had increased levels of Mre11, but also had higher levels of Rad50 and Nbs1 proteins, suggesting that the protein stability of Rad50 and Nbs1 depends upon complex formation with Mre11. Similar results were also obtained in an earlier study that transfected Mre11 into HCT116 cells [21]. Importantly, we also show that expression of Mre11 rescued SN38-induced S-phase arrest in HCT116 cells (Figure 1). We conclude that the failure of HCT116 cells to arrest in S phase on SN38 is due to their very low level of MRN.

### MRN complex protein levels vary greatly across the cell lines of the NCI60 panel

**Figure 2:**
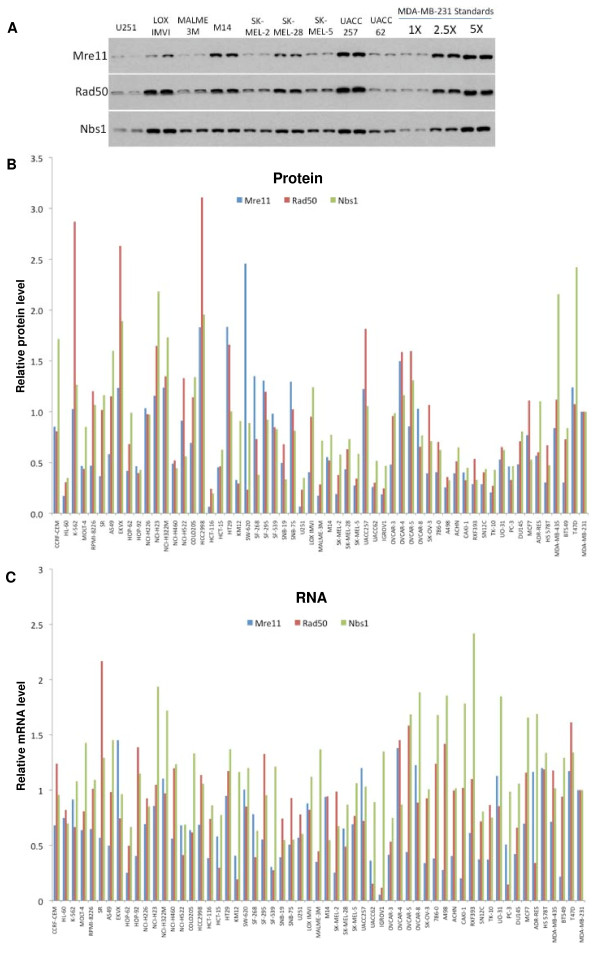
**MRN protein and mRNA levels in the NCI60 panel**. (A) Cell lines of the NCI60 panel were lysed and Mre11, Rad50, and Nbs1 proteins were analyzed by western blot. Lysates from each cell line were loaded in duplicate and three concentrations of MDA-MB-231 were loaded in duplicate on each gel. A representative blot is shown. (B) Multiple exposures of the western blots were scanned and quantified using Scion Image software. Protein levels were normalized to MDA-MB-231 = 1. (C) mRNA levels for Mre11, Rad50 and Nbs1 were derived from an Affymetrix human genome U133A-B array and obtained from the NCI Developmental Therapeutics Program database (http://dtp.nci.nih.gov; experiment ID# 223469). Values were normalized to MDA-MB-231 = 1. The numerical values for the data in panels B and C are provided as Table S1 in Additional file 1.

**Figure 3:**
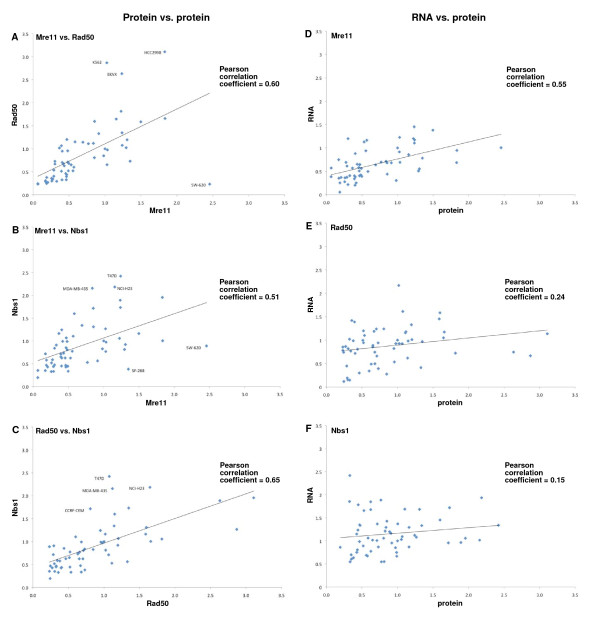
**Correlations between MRN proteins and mRNA levels**. (A) Mre11 protein levels in each cell line were graphed against (A) the Rad50 protein and (B) Nbs1 protein. (C) Rad50 protein was compared to Nbs1 protein levels. The cell lines with outlying values are identified. (D-F) Each protein was compared to its respective mRNA using the values in Figure 2.

The National Cancer Institute has screened more than 100,000 compounds in a panel of 60 cell lines and this data is publicly available. The database also has information on the gene expression patterns in each cell line. However, our data in Figure 1 suggests that MRN proteins are primarily regulated by stability so their levels are unlikely to be reflected by mRNA levels. In order to determine the frequency of defects in the MRN complex we made lysates from all of the cell lines. We assessed expression of Mre11, Rad50 and Nbs1 by western blot, and quantified the intensity of the protein bands (Figure 2A, B and Table S1 in Additional File 1). Expression of each protein was assessed relative to the levels in MDA-MB-231 which was set at a value of 1. There was a large range of expression with relative protein levels ranging from 0.06 to 2.4 for Mre11. There was also more than 10-fold difference in protein levels of Rad50 and Nbs1 (0.23 to 3.1 for Rad50, and 0.19 to 2.4 for Nbs1). There was a strong correlation between protein levels when we compared Mre11/Rad50, Mre11/Nbs1 and Rad50/Nbs1 (Figure 3A-C). This is consistent with our finding that transfection with Mre11 also increased Rad50 and Nbs1 levels. In addition to HCT116, a number of other cell lines expressed low levels of Mre11, in particular U251 had levels as low as HCT116. The next 5 lowest cell lines were HL60, MALME-3M, SK-MEL-2, IGROV1 and TK10. Each of these cell lines also had relatively low levels of Rad50 and Nbs1, consistent with the intact complex regulating the stability of each protein.

While this analysis was designed to identify cell lines with low levels of the MRN proteins, we also identified several lines in which the ratio of these proteins far exceeded the expected values. For example, SW620 cells had about 10 fold more Mre11 than Rad50, whereas Nbs1 was intermediate between these two levels. On the other hand, HCC2998, K562 and EKVX had about 2 fold more Rad50 than Mre11. For these latter cell lines, Mre11 and Nbs1 were relatively similar suggesting that it is the elevated level of Rad50 that maybe abnormal. Whether these cell lines contain functionally defective MRN remains to be determined.

To extend this analysis further, we compared the protein levels to mRNA levels obtained from the NCI database (Figure 2C and Table S1 in Additional File 1). The correlation coefficient for Mre11 mRNA and protein was 0.55, but only 0.24 and 0.15 for Rad50 and Nbs1, respectively (Figure 3D-F). This observation suggests that Mre11 protein levels are more highly regulated by mRNA levels whereas the Rad50 and Nbs1 protein levels are relatively independent of mRNA levels and likely depend more on protein stability mediated via formation of the MRN complex. It is also important to note that most of the cells we identified as having low levels of Mre11 protein had relatively normal levels of MRN mRNA suggesting that, like HCT116, they may contain a defective protein rather than the level of protein being regulated at the transcriptional level. The one notable exception was IGROV1 which has a very low level of Mre11 mRNA; surprisingly, these cells also exhibited a very low level of mRNA for Rad50. Therefore in this case, it appears that the reduced levels of the MRN complex are a consequence of transcription.

### Correlations between MRN protein levels and NCI60 compound activity

**Figure 4:**
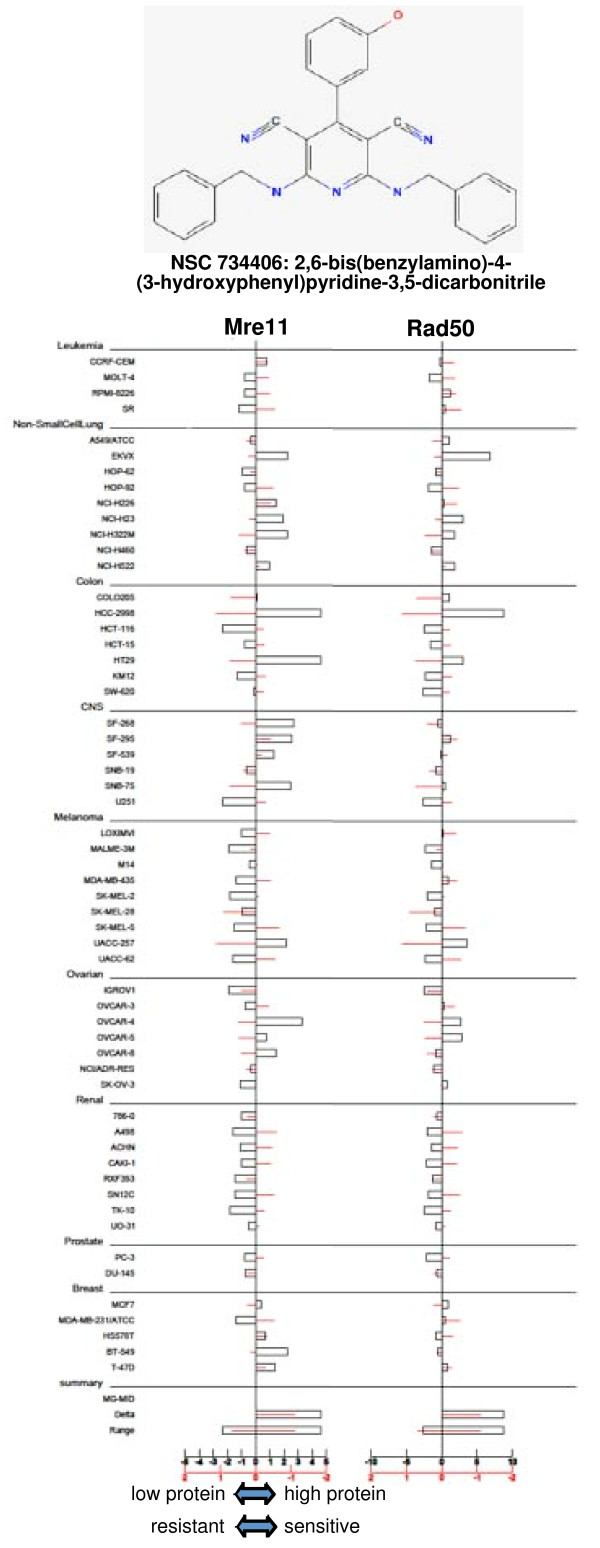
**Correlation of Mre11 and Rad50 protein levels with sensitivity to NSC 734406**. Relative protein levels (open bars) are compared to NSC 734406 sensitivity (red lines) in each cell line. The sensitivity data was obtained from http://dtp.nci.nih.gov. The center line in each data set reflects the mean for that parameter across the entire panel, and then the results for each cell line are represented to the left (low protein level or drug resistance) or to the right (high protein or drug sensitivity) of the mean. The observation that the red lines and boxes have an inverse correlation reflect the fact that low protein level correlates with drug sensitivity.

Considering that defective MRN can cause chemo- and radio-sensitivity, we hypothesized that other drugs in the NCI database would correlate with the low levels of MRN observed. Accordingly, we used the NCI COMPARE program to find correlations between MRN proteins and compound activity. We set the correlation coefficient to 0.5 to restrict the number of compounds identified. The search recovers both positive and negative correlations, that is, a positive correlation represents high protein level correlating with sensitivity whereas a negative correlation represents a cell line with low protein but high sensitivity to a compound. Mre11 and Nbs1 protein levels correlated with the activity of only a few compounds, and approximately equally distributed between positive and negative correlations (Table 1). In contrast, Rad50 protein levels correlated with many compounds, most of them positive. Three compounds were identified twice in the search, once each for Mre11 and Rad50 proteins. NSC 734406 correlated with low levels of Mre11 and Rad50 and therefore warrants validation and further study (Figure 4). The other two compounds, NSC359452 and NSC740487, correlated with high Mre11 and Rad50, but further examination of the dose response curves revealed the compounds were only marginally active in HCC2998 and HT29 cells and not in the other cell lines even at the highest concentration tested (10^-4 ^M). Hence, sensitivity to these latter compounds does not correlate with expression of the MRN proteins. This reflects a limitation of the COMPARE program that is discussed below.

**Table 1:** The highest correlating compounds for Mre11, Rad50 and Nbs1

Mre11		Rad50		Nbs1	
**Correlation**	**Compound**	**Correlation**	**Compound**	**Correlation**	**Compound**

0.542	NSC 654825	0.67	NSC 13728	0.527	NSC 640653

0.521	NSC 740847	0.622	NSC 686033	0.514	NSC 735029

0.517	NSC 647960	0.614	NSC 650947	0.509	NSC 704099

0.502	NSC 359452	0.605	NSC 371688	0.509	NSC 731647

		0.601	NSC 2353286	0.508	NSC 674948

					

-0.543	**NSC 734406**	-0.54	NSC 656576	-0.567	NSC 622089

-0.534	NSC 736184	-0.506	NSC 106320	-0.558	NSC 636870

-0.514	NSC 713292	-0.501	**NSC 734406**	-0.522	NSC 627268

-0.512	NSC 681130			-0.515	NSC 683903

					

Positive correlations >0.5 = 4		Positive correlations >0.5 = 44		Positive correlations >0.5 = 8	

Negative correlations >0.5 = 4		Negative correlations >0.5 = 3		Negative correlations >0.5 = 8	

**Bold **= NSC 734406 that was identified two times

### Lack of S-phase arrest in cell lines correlates with the activity of compounds, including several benzothiazoles

**Figure 5:**
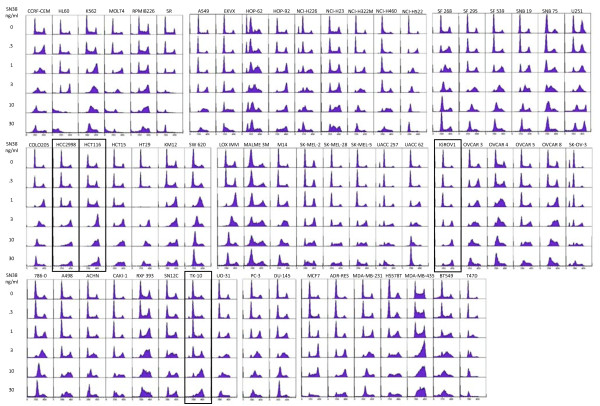
**Comparison of the ability of NCI60 cell lines to arrest in S-phase when incubated with SN38**. Cells were incubated with 0-30 ng/ml SN38 for 24 h then analyzed for cell cycle distribution by flow cytometry. The highlighted cell lines demonstrate little if any S arrest at the highest concentration.

**Figure 6:**
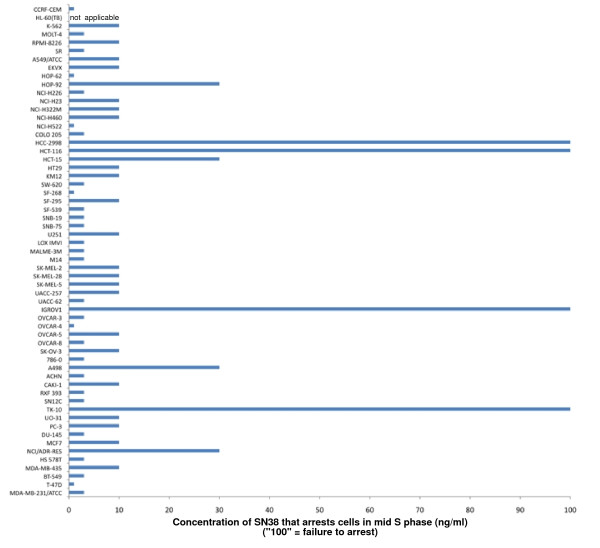
**Summary of the ability of NCI60 cell lines to arrest in S-phase when incubated with SN38**. Representation of the concentration of SN38 that arrested each cell line in mid-S phase as determined in Figure 5. The cell lines in which no arrest was observed are designated 100. HL60 cells underwent apoptosis rather than arrest so no number is assigned.

While the MRN complex has a critical role in the S-phase checkpoint, there are many other proteins that are also involved. In search of other possible S phase checkpoint defects, we screened all of the cell lines in the NCI60 panel for their ability to S-phase arrest in response to the topoisomerase I inhibitor SN38 (Figure 5). Several of the cell lines exhibited multiple peaks in untreated cells which is due to a mix of diploid and tetraploid cells. A mid S-phase arrest is observed in most of the cell lines when incubated with 3 - 10 ng/ml SN38. Several cell lines exhibited much more marked S-phase arrest at lower concentrations of SN38, for example T47D. One cell line, HL60, showed predominantly sub-G1 DNA content above 3 ng/ml indicative of apoptosis rather than arrest. Of the 59 cells lines tested, there were four that primarily arrested in G2 phase rather than S at the maximum concentration tested: HCT116, IGROV1, TK-10 and HCC2998 (these cell lines are designated "100" in Figure 6). Interestingly, the first three of these cell lines also had low MRN levels. For these cell lines, it is possible that the low MRN can explain their S-phase defect. In contrast, HCC2998 had one of the highest levels of Mre11 but lacked S-phase arrest. One possibility as discussed above is that the high Mre11 might reflect a functional defect in Mre11 and this would then explain their S-phase defect. However, several other cell lines, including U251 and HL-60, had low levels of MRN, but were still proficient for S-phase arrest, hence the level of MRN does not by itself predict whether a cell will arrest.

**Figure 7:**
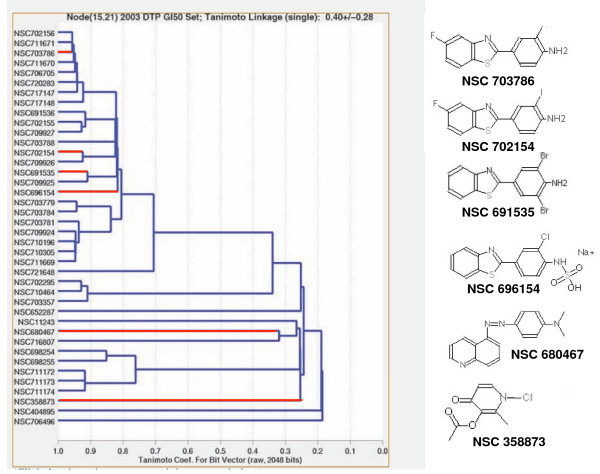
**Benzothiazoles highly correlate with lack of SN38-mediated S-phase arrest**. The k15.21 cluster from the 3dMind Map from the NCI60 database (http://spheroid.ncifcrf.gov/spheroid/). The compounds shown in red are in the top 30 correlations with lack of S- phase arrest.

**Figure 8:**
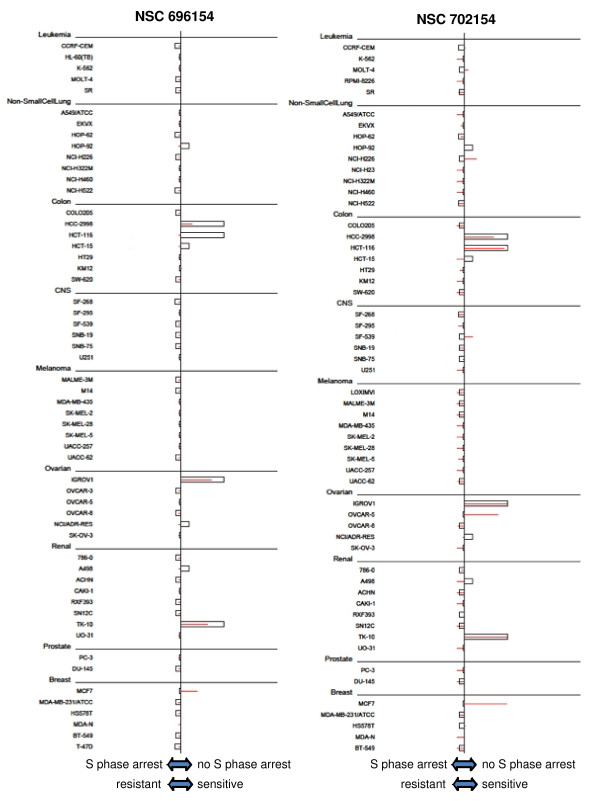
**Correlation of sensitivity to representative benzothiazoles and lack of SN38-mediated S-phase arrest**. The mean graphs for sensitivity to NSC 696154 (left panel, red lines) or NSC 702154 (right panel, red lines) are compared to the concentration of SN38 which causes S-phase arrest in each cell line (open bars). The observation that the red lines and boxes have a direct correlation reflect the fact that drug sensitivity correlates with a deficiency in S phase arrest.

**Figure 9:**
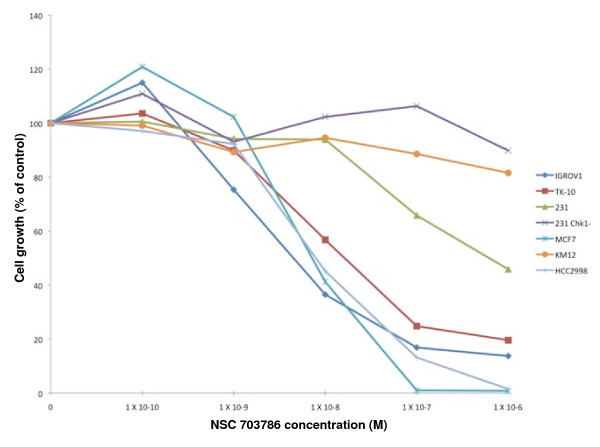
**Sensitivity of cells to the benzothiazole NSC 703786 (5F 203)**. Cells were incubated with 0 to 1 μM of NSC 703786 for 24 h. Drugs were removed and cells were grown for an additional 5-7 days. Cell growth was quantified by DNA content, as measured by Hoechst 33258 staining.

Using the COMPARE program to analyze the values in Figure 6, we identified 157 compounds that correlated with the lack of S arrest, but only 11 compounds that had a negative correlation (top 10 hits shown in Table 2). The correlation coefficients for the top compounds were much higher than those observed for correlations with MRN expression shown in Table 1. The top 30 compounds were analyzed individually. It was noted that many compounds exhibited poor correlation with the cell lines that failed to S-phase arrest, rather the relatively high correlation coefficients resulted from strong correlation with the other 55 cell lines rather than the 4 that failed to arrest. However, six of the top 30 correlations all derived from a single cluster (k15.21) of the 3dMind Map, a self organizing map which clusters similar cytotoxicity data into a two dimensional visual translation of related compounds (Figure 7). These clusters generally reflect compounds with a similar mechanism of action, although cluster k15.21 is unrelated to any common anticancer mechanism. About half of the compounds in this cluster, have closely related structures and are known as benzothiazoles, although other structures such as NSC 680467 also showed high correlation with cells that failed to S phase arrest. The correlation with S phase arrest for two of the benzothiazoles is shown in Figure 8. In general, most of the compounds in cluster k15.21 inhibit growth of 5 cell lines, HCC2998, IGROV-1, TK10, MCF7 and T47D (although the latter is not evident for the compounds shown in Figure 6B), but only the first three of these failed to arrest in S phase when incubated with SN38. To validate these results, we obtained the benzothiazole NSC 703786 (5F 203; the only one available from NCI) and assessed growth inhibition in various cell lines including those that lack S-phase arrest (Figure 9). Our results were consistent with those from the NCI database.

**Table 2:** Compounds with the highest correlation with SN38-induced S phase arrest

SN38-induced S phase arrest	
**Correlation**	**Compound**

0.82	NSC 691535

0.791	NSC 649554

0.752	NSC 680467

0.726	NSC 358873

0.721	NSC 648601

-0.7	NSC 641318

-0.65	NSC 677599

-0.644	NSC 615291

-0.592	NSC 633267

-0.541	NSC 638404

Positive correlations >0.5 = 157	

Negative correlations >0.5 = 11	

The sensitivity of cells to benzothiazoles is reported to be due to the ability of cells to metabolically activate these compounds which then go on to damage DNA [22,23]. MCF7 cells exhibit the highest metabolism of benzothiazoles and we therefore anticipated that they should sensitize non-responsive cells in co-culture. We used transwell plates in a 96 well format and plated ~50,000 MCF7 cells in the upper well. Other cell lines, including HCT116, were plated at low density in the lower wells. NSC703783 (1 μM) was added for 24 h then removed, in an experiment similar to that in Figure 9. The MCF7 cells had no impact on the growth of six cell lines tested. We conclude that differences in metabolism do not adequately explain the differences in sensitivity of cells to the benzothiazoles.

## Discussion

The overall goal of this study was to identify molecular defects in cell cycle checkpoint regulation using the NCI60 panel of cell lines, and then to identify compounds that might target cells with these defects. Eventually, such compounds might provide leads for the development of drugs for personalized cancer therapy. We began by screening the cell panel for potential defects in MRN proteins and the S-phase checkpoint. We uncovered a number of interesting relationships with respect to MRN proteins and their mRNA that impact the level of this complex. In addition, using the COMPARE program, we have identified several compounds of interest which warrant further study.

Our results implicate protein stability in the regulation of MRN protein levels. Transfection with Mre11 not only increased Mre11 levels but also Rad50 and Nbs1 levels. In comparing the protein levels in each cell line across the NCI60 panel, we also found a strong correlation between the levels of these three proteins. The overall level of the complex appeared to be primarily regulated by transcription of Mre11 as there was much lower correlation between mRNA and protein levels for Rad50 and Nbs1. These results suggest that the protein levels of Rad50 and Nbs1 are primarily regulated by stability of the complex while transcription of Mre11 contributes more to the overall level of the complex.

While Mre11 expression may be more highly regulated by transcription, among the cell lines with low levels of Mre11 protein, only the IGROV1 cells had a very low level of mRNA. HCT116 had a relatively high level of Mre11 transcript but low protein, and this has been shown to be due to a splicing defect in its mRNA [9]. This defect occurs as a frame shift in a poly(dA) track in an intron which is consistent with these cells being defective for mismatch repair MLH1 [9,24]. Five cell lines in the NCI60 panel are defective for MLH1 [25], one of which is IGROV1, but the other three (SKOV3, KM12 and CCRF-CEM) still express relatively normal levels of Mre11. None of the NCI cell lines are defective in the mismatch gene MSH2. Hence mismatch repair defects do not appear to explain the low levels of Mre11 protein in these other cell lines. Other mutations which might result in protein degradation could be a possible cause for the low protein levels in the other cells but further study is necessary. In addition, there were several cell lines with unbalanced levels of MRN, for example, unexpected high or low levels of Rad50 compared to Mre11. One possible explanation for this observation is that the proteins contain a mutation which confers greater protein stability when they are not in the MRN complex.

This screen identified a small number of compounds which correlated with either Mre11, Rad50, or Nbs1. However, there were three compounds identified which correlated with both Mre11 and Rad50. The activity of one of these, NSC 734406, was significant at the concentrations tested and correlates with low levels of Mre11 and Rad50 (Figure 4). Validation and further study with this compound is necessary to assess whether it does preferentially kill cells with low MRN protein but this was not possible as the compound is not available from the NCI repository. One of the potential limitations of this COMPARE analysis is that protein levels may not necessarily reflect MRN function, particularly in those cases where MRN protein expression is unbalanced. Another approach would be to assay the cells for MRN activity using a functional readout, such as Chk1 phosphorylation in response to ionizing radiation, for which the MRN complex is required [26]. Alternately the recruitment of ATM to damaged DNA could be assayed as this also depends on the MRN complex.

**Figure 10:**
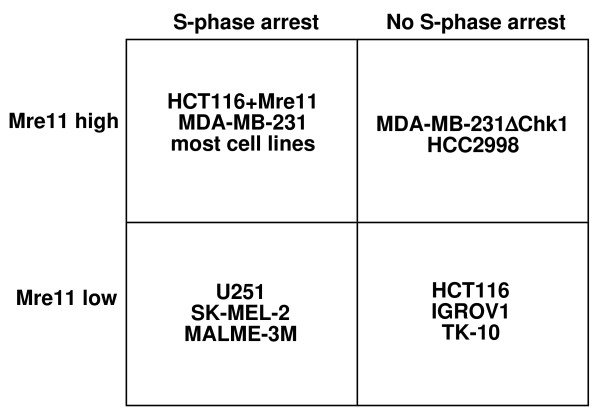
**The relationship between MRN protein expression and ability of cells to arrest in S phase when incubated with SN38**.

In searching the NCI60 panel for cell lines with defects in the S-phase checkpoint we identified three cell lines, in addition to HCT116, which failed to arrest in S-phase in response to SN38. HCT116, TK-10 and IGROV1 all have low levels of the MRN which may explain their S phase defect (see summary in Figure 10). In contrast, HCC2998 cells fail to arrest in S phase yet have very high levels of Rad50 and Mre11 proteins. It remains to be determined whether the MRN complex in this cell line is functional. If functional, this would suggest that while the MRN complex is necessary for S-phase arrest, it is not sufficient. There were also several cell lines with low levels of the MRN proteins that were proficient for S-phase arrest. A possible explanation is that low levels of MRN maybe sufficient for S-phase arrest but that a mutation in Mre11 as occurs in HCT116 may be necessary to elicit the checkpoint the defect. For example, it has been suggested that the mutant form of Mre11 in HCT116 acts in a dominant negative manner and this may be far more detrimental than simply having low levels of Mre11 [24].

We have previously reported an alternate means to prevent S phase arrest [27]. The expression of shRNA against Chk1 in MDA-MB-231 cells overcame the S phase arrest induced by SN38, although such cells still arrested in G2. We observed a similar response when cells were co-incubated with SN38 and a small molecule inhibitor of Chk1. There is a close link between the MRN complex and Chk1 activation. Specifically, MRN is recruited to double-strand breaks where the exonuclease activity of Mre11 creates single-stranded DNA that, through a series of events, recruits ATR and activates Chk1. However, it has been shown that HCT116 cells are fully competent to activate Chk1 when damaged with topoisomerase I inhibitors [21] and we have confirmed this in our own laboratory (data not shown). This is likely explained by the topoisomerase I inhibitors stalling replication fork progression thereby activating ATR and Chk1 without the need to implicate ATM and Mre11. This raises the conundrum as to why SN38 is fully capable of activating Chk1 in HCT116 cells yet the cells fail to S phase arrest unless they are complemented with Mre11. The results suggest that activation of Chk1 may be necessary but not sufficient to induce S phase arrest and that the MRN complex plays some additional role.

In searching for correlations between S-phase checkpoint defects and compound activity, we identified many drugs which correlated positively, meaning these compounds have activity in cell lines with defects. Six compounds out of the top 30 correlations identified mapped to the same cluster indicating that they share the same mechanism of growth inhibition. Four of these are benzothiazoles, a class of compounds that has been shown to be metabolized by sensitive cells and then form DNA adducts [22,23]. Whether this is also true of other compounds in this cluster has not been determined. However, we note that KM12 cells have previously been shown to metabolize benzothiazoles, yet they are insensitive to these compounds [28]. Similarly we note that TK10 cells are as sensitive as MCF7, yet have markedly lower level of metabolism [22]. The lack of sensitivity of HCT116 to benzothiazoles may be due to their inability to metabolize the compound, however HCT116 cells (and many others) remained insensitive to benzothiazoles when co-cultured with MCF7 cells. Accordingly, while metabolism may contribute to the sensitivity to these compounds in some cells, there may also be a major contribution from the S-phase checkpoint defect.

The fact that the S-phase checkpoint search was a functional assay provides greater confidence in the candidates identified than with the MRN protein screen. However, because the S-phase defect can be caused by multiple reasons (e.g., low or defective ATM, ATR, Mre11 or Chk1), we may have missed many potential drug candidates. Some compounds may correlate with only some of the checkpoint defects responsible for the lack of SN38-induced S-phase arrest. The fact that each defect may be rare in the NCI60 panel makes it a challenge to use this approach to identify novel drug candidates. For example, a compound that has the same activity in 60 cell lines would exhibit a very high correlation with a dataset in which 56 cell lines arrested and 4 did not, the correlation coefficient being driven by the 56 cell lines rather than the 4 interesting queries. Accordingly, each compound discovered in the screen had to be individually analyzed and validated. Many compounds elicited no obvious correlation with the query set. In some cases (e.g., NSC358873), three of the four cell lines that failed to arrest in S phase had not even been screened against this compound. These limitations are unfortunate because we had hoped to use this approach to identify compounds that killed only a few cell lines, and might therefore be candidates for selective therapy for tumors with that particular phenotype (i.e., individualized therapy). Given the limitations of this screen, the fact that most of the cells defective for S phase arrest were indeed sensitive to compounds in the benzothiazole cluster gives strong support for further investigation into this class of drugs.

## Conclusions

Genomic instability is one of the characteristics of cancer and is frequently attributable to defects in DNA damage response genes involved in either DNA repair or checkpoint regulation. This provides a therapeutic opportunity as these defective cells may be hypersensitive to various drugs. We have identified cells with potential defects in the MRN complex and in S phase arrest, and a series of compounds that may preferentially target S phase arrest-defective cells. The in silico screen used has limitations when seeking compounds that are cytotoxic to only a few cell lines. But considering this limitation, the identification of benzothiazoles as compounds that preferentially target cells with a defect in S phase arrest warrants further investigation. Compounds such as these that kill only a few cell lines likely represent the future of individualized cancer therapeutics.

## Competing interests

The authors declare that they have no conflicting interests

## Authors' contributions

KMG and AE conceived the project and shared in its design. KMG performed most of the experiments. KMG and AE performed data analysis and wrote the manuscript. Both authors read and approved the final manuscript.

## Pre-publication history

The pre-publication history for this paper can be accessed here:

http://www.biomedcentral.com/1471-2407/11/206/prepub

## Supplementary Material

Additional file 1 **Table S1: Correlations between Mre11, Rad50 and Nbs1 protein and mRNA**. Click here for file
