# Supplementary material for: Variations in Mre11/Rad50/Nbs1 status and DNA damage-induced S-phase arrest in the cell lines of the NCI60 panel
Source: BMC Cancer. 2011 May 27;11:206. doi: 10.1186/1471-2407-11-206 (PMC3128005; doi:10.1186/1471-2407-11-206)
Supplement: Additional file 1 — Table S1: Correlations between Mre11, Rad50 and Nbs1 protein and mRNA. [file 1471-2407-11-206-S1.PDF]

Table S1. Correlations between Mre11, Rad50 and Nbs1 protein and mRNA. Cell lines of the NCI60 panel were lysed and analyzed for expression of Mre11, Rad50 and Nbs1 as shown in Figure 2. Western blots were scanned and levels of each protein were compared to a dilution series of MDA-MB-231. mRNA levels were obtained from the NCI Developmental Therapeutics Program database (http://dtp.nci.nih.gov; experiment ID# 223469). Expression levels were normalized to MDA-MB-231 = 1, for each protein. The values presented in this table are graphically represented in Figure 2 of the paper.

	PROTEIN			mRNA		
**CELL LINE**	**MRE11**	**RAD50**	**NBS1**	**MRE11**	**RAD50**	**NBS1**
CCRF-CEM	0.85	0.81	1.72	0.68	1.24	0.96
HL-60	0.17	0.31	0.35	0.75	0.82	0.70
K-562	1.03	2.87	1.27	0.92	0.67	1.08
MOLT-4	0.47	0.44	0.85	0.64	0.81	1.43
RPMI-8226	0.47	1.20	1.07	0.65	1.01	1.09
SR	0.37	1.02	1.16	0.57	2.17	1.29
A549	0.58	1.15	1.60	0.50	0.98	1.45
EKVX	1.23	2.63	1.89	1.45	0.74	0.96
HOP-62	0.42	0.68	0.99	0.25	0.50	0.67
HOP-92	0.46	0.40	0.43	0.40	1.39	1.15
NCI-H226	1.03	0.98	0.97	0.69	0.93	0.85
NCI-H23	1.16	1.65	2.18	0.86	1.05	1.94
NCI-H322M	1.24	1.35	1.73	1.10	0.97	1.72
NCI-H460	0.49	0.52	0.44	0.56	1.20	1.23
NCI-H522	0.91	1.33	0.56	0.68	0.41	0.69
COLO205	0.69	1.14	1.34	0.64	0.62	1.33
HCC2998	1.83	3.11	1.96	0.69	1.14	1.06
HCT-116	0.07	0.24	0.19	0.38	0.74	0.86
HCT-15	0.45	0.46	0.63	0.58	0.30	0.78
HT29	1.84	1.66	1.00	0.95	1.17	1.37
KM12	0.33	0.29	0.91	0.41	0.19	1.16
SW-620	0.63	0.23	0.89	1.00	0.85	1.20
SF-268	1.35	0.73	0.38	0.78	0.39	0.63
SF-295	1.31	1.20	0.92	0.55	1.33	0.95
SF-539	0.98	0.85	0.83	0.30	0.27	1.21
SNB-19	0.50	0.68	0.33	0.39	0.74	0.55
SNB-75	1.29	1.02	0.81	0.51	0.93	0.55
U251	0.07	0.23	0.35	0.57	0.78	0.60
LOX IMVI	0.41	0.95	1.24	0.88	0.82	1.12
MALME-3M	0.17	0.29	0.72	0.35	0.45	1.37

M14	0.55	0.52	0.77	0.94	0.94	0.55
SK-MEL-2	0.19	0.38	0.58	0.25	0.99	0.67
SK-MEL-28	0.43	0.63	0.73	0.65	0.49	0.87
SK-MEL-5	0.27	0.34	0.59	0.69	0.77	1.06
UACC257	1.22	1.81	1.06	1.20	0.72	1.03
UACC62	0.26	0.30	0.52	0.36	0.15	0.89
IGROV1	0.19	0.25	0.47	0.05	0.12	1.35
OVCAR-3	0.48	0.96	0.99	0.42	0.53	0.75
OVCAR-4	1.50	1.59	1.16	1.38	1.45	0.87
OVCAR-5	0.86	1.60	1.31	0.44	1.58	1.68
OVCAR-8	1.03	0.66	0.77	1.23	0.89	1.89
SK-OV-3	0.39	1.07	0.71	0.34	0.93	1.01
786-0	0.41	0.70	0.62	0.38	1.24	1.68
A498	0.26	0.36	0.33	0.28	1.42	1.86
ACHN	0.39	0.51	0.65	0.40	1.00	1.02
CAKI-1	0.40	0.33	0.45	0.20	1.02	1.78
RXF393	0.29	0.54	0.33	0.61	1.10	2.42
SN12C	0.29	0.40	0.44	0.37	0.72	0.81
TK-10	0.21	0.27	0.43	0.37	0.87	0.75
UO-31	0.53	0.65	0.62	1.13	0.85	1.85
PC-3	0.46	0.33	0.47	0.51	0.15	0.99
DU145	0.48	0.71	0.81	0.42	0.66	1.06
MCF7	0.77	1.11	0.53	0.70	1.16	1.66
ADR-RES	0.57	0.60	1.10	1.16	0.34	1.69
HS 578T	0.30	0.67	0.47	1.20	1.19	1.34
MDA-MB-435	0.84	1.12	2.16	0.71	1.18	1.02
BT549	0.30	0.73	0.84	0.22	0.94	1.29
T47D	1.24	1.08	2.42	1.17	1.61	1.34
MDA-MB-231	1.00	1.00	1.00	1.00	1.00	1.00
